# Supplementary material for: Divergent proinflammatory immune responses associated with the differential susceptibility of cattle breeds to tuberculosis
Source: Front Immunol. 2023 Sep 19;14:1199092. doi: 10.3389/fimmu.2023.1199092 (PMC10546398; doi:10.3389/fimmu.2023.1199092)
Supplement: Supplementary file 1 [file DataSheet_1.pdf]

Supplementary material

Divergent proinflammatory immune responses associated with the differential susceptibility of cattle breeds to tuberculosis

Authors:

Rishi Kumar[1,2], Sripratyusha Gandham[1,2], Avi Rana[1], Hemanta Kumar Maity[3], Uttam Sarkar[4], Bappaditya Dey[1,2*]

Affiliations:

[1]National Institute of Animal Biotechnology, Hyderabad, Telangana, India, PIN 500032

[2]Regional Centre for Biotechnology, Faridabad, Haryana, India, PIN 121001

[3]Department of Avian Sciences, West Bengal University of Animal and Fishery Sciences. Kolkata, West Bengal, India, PIN 700037.

[4]Department of Animal Genetics and Breeding, West Bengal University of Animal and Fishery Sciences. Kolkata, West Bengal, India, PIN 700037.

***Corresponding Author:**

Dr. Bappaditya Dey
Scientist-E
National Institute of Animal Biotechnology
Hyderabad, Telangana, India - 500032
Telephone: 040-23120128
Email ID: bdey@niab.org.in

Supplementary Figure S1

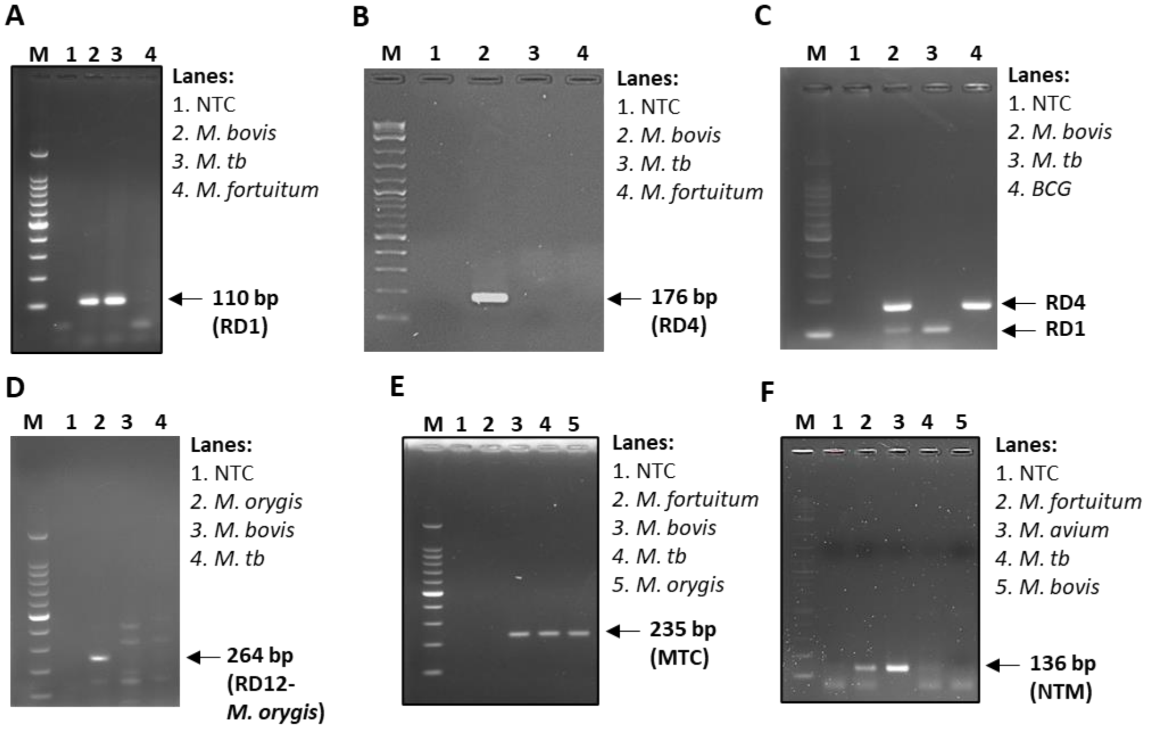

Supplementary Figure S1. PCR-based detection of mycobacterial DNA. Representative agarose gel electrophoresis images of the PCR analysis targeting specific genomic regions of mycobacteria to detect (A) RD1 region- positive for *M. tuberculosis* and *M. bovis*, (B) RD4 region- positive of *M. bovis*, (C) RD1 and RD4 combined region to differentiate *M. tuberculosis*, *M. bovis,* and BCG, (D) RD12- *M. orygis,* (E) pan MTC, and (F) pan- NTM. Genomic DNA from different species of mycobacteria was used as the positive template for PCR. NTC- non-template control.

Supplementary Figure S2

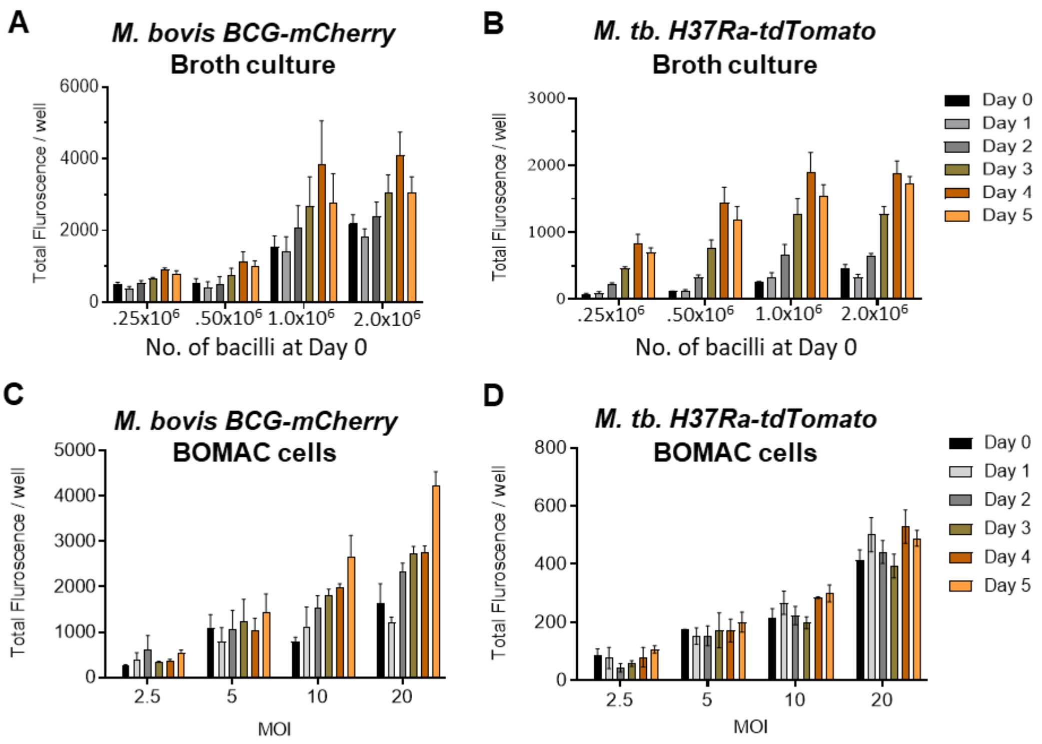

Supplementary Figure S2. Association of fluorescent reporter mycobacterial number with the total fluorescence. The association of the fluorescence of the reporter mycobacterial strains- *M. bovis* BCG-mCherry and *M. tuberculosis H37Ra-tdTomato* expressing mCherry and tdTomato to the CFU were evaluated both in the (A, B) 7H9 broth culture and (C, D) BOMAC cells, respectively. The bacterial growth rate in 7H9 broth was determined by plotting the total fluorescence intensity/well against the different numbers of bacteria: 0.25 M, 0.5 M, 1 M, and 2 million at day-0 of the culture, and monitored for 5 days. For growth in the BOMAC cells, cells were infected with the reporter strains at different MOI (2.5, 5, 10, and 20) and the fluorescence intensity was monitored every day for 5 days post-infection. The fluorescence intensity was measured at λex/ λem 554/581nm (*M. tuberculosis H37Ra-tdTomato*), and 587/610nm (*M. bovis BCG*-mCherry). The Pearson's correlation coefficient (*r*) between the bacterial CFU and fluorescence intensity was found to be strongly positive (r = 0.874-0.967, and 0.841-0.990 for *M. tuberculosis H37Ra-tdTomato* in 7H9 broth and BOMAC culture, respectively over 5 days; r = 0.780-0.990, and 0.974-0.999 for *M. bovis BCG-mCherry* in 7H9 broth and BOMAC culture, respectively). An MOI of 1: 10 was considered for *ex vivo* mycobacterial growth in the bPBMC.

Supplementary Figure S3

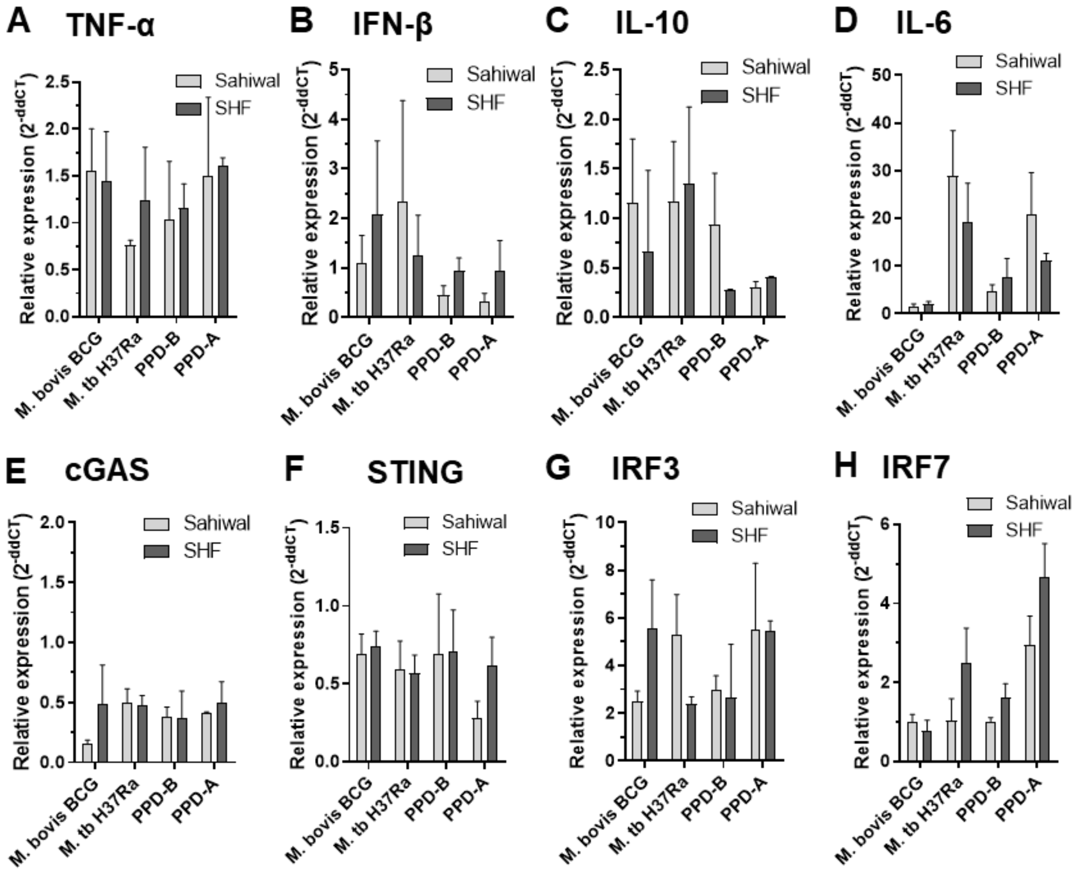

Supplementary Figure S3. Host immune responses-related gene expression profiling in bovine PBMC upon mycobacterial infection and antigenic stimulation. Expression of various cytokines and immunity-related genes were measured on the RNA extracted from PBMC infected with M. bovis BCG or M. tuberculosis H37Ra at an MOI of 1:10 (cell: bacteria) or stimulated with bovine PPD (PPD-B, 300 IU/ml) and Avium PPD (PPD-A, 250 IU/ml at 24-hour post-infection by semi-quantitative real-time RT-PCR using gene-specific primers. (A) TNF-α, (B) IFN-β, (C) IL-10, (D) IL-6, (E) cGAS, (F) STING, (G) IRF3, and (H) IRF7. No difference was observed between the two breeds. The data were normalized to RPLP0 expression levels and then normalized to the values of uninfected/unstimulated cells to obtain ddCT values. Data is represented as a bar diagram of mean ± SEM of 2^{-ddCT} values as relative expression, n = 3, *, $p<0.05$ (t-test). The data are representative of two experiments.

Supplementary Table S1: Animal-wise distribution of SITT and PCR positivity.

Animal code No	RD1+	RD4+	MTC+	NTM +	SITT+	SITT+ RD1/RD4/ MTC+	SITT+ or RD1/RD4/ MTC +	SITT+ RD1/RD4/ MTC -	SITT- RD1/RD4/ MTC +	Any positive
S1										
S2			+	+	+	+	+			+
S3				+	+		+	+		+
S4										
S5										
S6										
S7										
S8										
S9										
S10										
S11										
S12										
S13										
S14										
S15										
S16										
S17										
S18										
S19										
S20										
S21										
S22										
S23										
S24										
SHF1			+	+			+		+	+
SHF2	+	+	+	+			+		+	+
SHF3				+						+
SHF4				+						+
SHF5	+		+	+	+	+	+			+
SHF6	+		+	+			+		+	+
SHF7			+	+			+		+	+
SHF8				+						+
SHF9	+	+		+			+		+	+
SHF10	+						+		+	
SHF11										
SHF12			+		+	+	+			+
SHF13										
SHF14										
SHF15										
SHF16										
SHF17										
SHF18					+		+	+		+
SHF19					+		+	+		+
SHF20					+		+	+		+
SHF21				+	+	+	+			+
SHF22				+	+	+	+			+
SHF23				+						+
SHF24										
SHF25				+						+
SHF26				+						+
Total	5	2	7	16	9	5	15	4	6	20

+, positive of the given type; Blank cell, negative for the given type

Supplementary Table S2: PCR primers used for mycobacterial detection		
**Name**	**Nucleotide Sequence**	**Reference**
RD1	F, 5'-CCCTTTCTCGTGTTTATAGTTTGA-3'<br>R, 5'-GCCATATCGTCCGGAGCTT-3'	27
RD4	F, 5'-AATGGTTTGGTCATGACGCCTTC-3'<br>R, 5'-CCCGTAGCGTTACTGAGAAATTGC-3'	26
RD12-<br>*M. orygis*	F, 5'-GTGGAAATGGAAGCGTTGACC-3'<br>R, 5'-GGTACCTCCTCGATGAACCAC-3'	25
MTC	F, 5'-CGTACGGTCGGCGAGCTGATCCAA-3'<br>R, 5'-CCACCAGTCGGCGCTTGTGGGTCAA-3'	24
NTM	F, 5'-GGAGCGGATGACCACCCAGGACGTC-3'<br>R, 5'-CAGCGGGTTGTTCTGTCCATGAAC-3'	24

**Supplementary Table S3: Plasmids, mycobacterial strains, and mycobacterial antigens/cell components.**		
**Name**	**Description**	**Source**
**Plasmids**		
pMSP12::mCherry	Mycobacterial reporter plasmid expressing mCherry	Addgene plasmid # 30169
pTEC27-Hyg	Mycobacterial reporter plasmid expressing tdTomato	Addgene plasmid # 30182
**Mycobacterial strains**		
M. tuberculosis H37Ra	*M. tuberculosis* H37Ra strain- *BSL2 grade laboratory strain*	*Prof. Sharmistha Banerjee, University of Hyderabad*
M. bovis BCG	*M. bovis BCG Danish 1331 strain*	NIBSC, UK
M. tuberculosis H*37Ra*-tdTomato	*M. tuberculosis* H37Ra strain- *harbouring* pTEC27-Hyg	*This study*
M. bovis BCG - mCherry	*M. bovis BCG Danish 1331* strain harboring pMSP12::mCherry	*This study*
M. fortuitum	Non-tuberculous mycobacterium strain	*MTCC repository, CSIR-IMTECH, India*
**Mycobacterial antigens/cell components**		
PPD-A	Purified protein derivative from *M. avium*	*ThermoFisher*
PPD-B	Purified protein derivative from *M. bovis*	*ThermoFisher*
WCL	*M. tuberculosis whole cell lysate*	*BEI Resources, USA*
CW	*M. tuberculosis cell wall*	*BEI Resources, USA*
LAM	*M. tuberculosis Lipoarabinomannan*	*BEI Resources, USA*

Supplementary Table S4: Real-time PCR primers (bovine genes)		
**Primer Name**	**Nucleotide Sequence**	**Gene description**
*bIFN-γ	F, 5′-gctgattcaaattccggtgga-3′<br>R, 5′-caggcaggaggaccattacg-3′	Interferon-gamma
bIL-17A	F, 5′-agaaggcccaccgattatca-3′<br>R, 5′-ccaccttcccttcagcattga-3′	Interleukin 17
bTNF-α	F, 5′-accagccaggagagagacaa-3′<br>R, 5′-gatcatgcttttggtgctca-3′	Tuner Necrosis Factor Alfa
bIL-1β	F, 5’-cagtgcctacgcacatgtct-3′<br>R, 5′-ccagggatttttgctctctg-3′	Interleukin 1 beta
bIL-6	F, 5′-cagctatgaactcccgcttc-3′<br>R, 5′-ttcggttttctctggagtgg-3′	Interleukin 6
bIL-10	F, 5′- tgttgacccagtctctgctg-3′<br>R, 5′- agcttctcccccagtgagtt-3′	Interleukin 10
bcGAS	F, 5′-ttcaaaggcgtagacctgct-3′<br>ccgataagattccccttct-3′	Cyclic GAMP synthase
bSTING	F, 5′-tgcatccatccatcccacag-3′<br>R, 5′-caccagacaggcacttagca-3′	Stimulator of interferon genes
bTBK1	F, 5′-tgcagctactggatcactgc-3′<br>R, 5′-aacaggcatgtctccactcc-3′	Tank binding kinase 1
bIRF3	F, 5′-aagccccacctctaaagctc-3′<br>tatcagccagggcagtatcc-3′	Interferon regulatory factor 3
bIRF7	F, 5′-gcctcctggaaaaccaactt-3′<br>R, 5′-atcttctagggcctcgtcct-3′	Interferon regulatory factor 7
bIFN-β	F, 5′-actcctggggcagttacctt-3′<br>R, 5′-ctggtgagaatgccgaagat-3′	Interferon-beta
bRPLP0	F, 5′-cttcattgtgggagcagaca-3′<br>R, 5′-ggcaacagtttctccagagc-3′	60S acidic ribosomal protein large
bGAPDH	F, 5′-atctctgcaccttctgccga-3′<br>R, 5′-gcaggaggcattgctgaca-3′	glyceraldehyde-3-phosphate dehydrogenase
*b, Bovine.		
